# An Introductory Lecture on the Means of Promoting the Intellectual Improvement of the Students and Physicians of the Valley of the Mississippi

**Published:** 1845-03

**Authors:** 


*An Introductory Lecture on the Means of Promoting the Intellectual Improvement of the Students and Physicians of the Valley of the Mississippi. Delivered in the Medical Institute of Louisville, November 4th,* 1844. By Daniel Drake, M. D., Professor of Pathology and the Practice of Medicine. Louisville, Ky., 1844.

I appears that by a late regulation of the Institute, but one Introductory Lecture is delivered at the opening of each session. The present, therefore, is not an introductory to the course on the particular branch taught by the Professor, but is made to embrace whatever subjects of interest the author deemed appropriate to the occasion. It is chiefly ethical in its character, and conveys much sound instruction and good advice as to the general conduct of Students about to enter upon a Winter's course of lectures, and happy will it be for those before whom it was delivered, if its precepts shall be remembered and obeyed by them.

